# It’s time to eliminate the mismatch between pediatric training and practice

**DOI:** 10.1186/s13584-021-00504-7

**Published:** 2021-12-02

**Authors:** Frank Oberklaid

**Affiliations:** Centre for Community Child Health, Royal Children’s Hospital Melbourne, Murdoch Children’s Research Institute, Melbourne, Australia

## Abstract

The quality of pediatric clinical practice is dependent on the training received during residency. It is assumed that the content of the training will adequately prepare pediatricians for the sorts of problems and issues they will be asked to manage in community settings. While over the past several decades there have been major changes in pediatric morbidity, there is evidence that training and service delivery models have not evolved; there is a significant mismatch between training and evidence-based clinical practice. A recent paper published in this journal (1) drew attention to the inadequacy of pediatricians’ training in child development. The reality of major gaps in the content and experiences of pediatric training in Israel are widely held, and there have been repeated calls for an increased focus on community child health and developmental and behavioural pediatrics. While it appears that finally there are some small initial steps to this end, it is strongly recommended that there be a long overdue, radical rethink of pediatric training programs.

## Closing the gap: the mismatch between pediatric training and pediatric practice in Israel

It is self-evident that the training of pediatricians must equip them with the knowledge and skills to be able practice at a high level of competence when they finish their training and enter clinical practice. It is inconceivable that the pediatric subspecialties would not have a formal training program in that specialty and that knowledge and expertise would be expected to be learned ‘on the job’ and by ad hoc, unsupervised rotations to outpatient or community settings. Yet that is exactly the situation that exists for large numbers of Israeli pediatricians.

In a recent paper published in this journal [1]. Latzer and his colleagues reported on a study of Israeli pediatricians where they assessed their knowledge of child development and associated areas. They asked the pediatricians whether they felt their training in child development had been adequate. Their knowledge of child development-related topics, as tested by a series of questions on a specially developed knowledge survey, was disappointingly low. A staggering 94% of pediatricians in their sample rated their training in child development as inadequate, while 80% felt they had insufficient access to guidelines and resources, and two thirds felt they needed to strengthen their knowledge of child development topics. The Israeli institutions and authorities will need to decide whether this is an acceptable state of affairs; I would argue that it is not.

Parents often have concerns about their child’s development and/or behavior; pediatricians need to have the skills and confidence to assess the severity of these problems and take appropriate action. This can be simply reassuring parents that all is well, providing advice for management of (for example) behavior problems, or making an informed referral for further assessment. Without adequate training, there is a risk of pediatricians giving false reassurance to parents, or continuing to overload tertiary referral centres with problems that could have been managed in the pediatric office. In addition to adding to parents’ anxiety and distress, the opportunity for timely and effective interventions may be missed.

In Israel ‘*the official academic syllabus of the residency states that certified pediatricians should be knowledgeable of basis subject matters related to child development*…’1). However, there is no designated period of training during residency while there is a mandated 6 months training in neonatal intensive care. It is interesting to reflect on how many pediatricians will be expected to look after sick hospitalized infants in their future clinical practice. It is assumed that trainees’ knowledge of child development will be somehow gained during their training ‘…*from senior pediatricians…outpatient clinics and on the job learning in randomly assigned daily shifts …in community based well baby clinics*.’[1]

Calls for increased training in child development are not new. Richmond, in his seminal 1967 paper [2], argued that child development should be considered a basic science for pediatrics, and that ‘*knowledge of child development is a major concern of the practising pediatrician*.’ That same year Eisenberg drew attention to the imbalance of pediatric training programs [3]. ‘*Pediatrics has long neglected its responsibility for the study of child development…academic pediatrics has lagged in its interest in the non-hospitalized child… it prepares its house officers exceedingly well for what they will rarely see and says hardly a word about what will confront them daily*.’ In 1975 Haggerty, considered the grandfather of community child health, coined the term ‘the new morbidity' to describe the changing patterns of children’s health issues [4]. ‘*A group of childhood difficulties that we have termed ‘the new morbidity is now gaining attention. Many of these difficulties lie beyond the boundaries of traditional medical care… Handling such problems will be important to the future of pediatric practice, and a major shift in the orientation of training programs is required to prepare pediatricians for these tasks*.’ In the USA the response—many years later—was to establish a Task Force to ‘…*consider the health needs of American children and how the educational process may better equip pediatricians and other child health care professionals to meet those needs’* [5]*.*

In Australia the changes in pediatric practice have been well documented [6, 7] with national surveys confirming that developmental and behavioural issues are the most common problems seen by pediatricians. In Israel [8] as well as in Australia [9] pediatricians have pointed to deficiencies in their training which they feel has not adequately prepared them for the sorts of problems they are asked to see.

The case for a focus on child development has become even more compelling with our increased understanding of the importance of the early years and the risk factors that threaten optimal health and development. A voluminous body of work tells us that the first few years of a child’s life are critical in impacting on outcomes right through the life course into adult life [10], and there is robust research pointing to the powerful negative influence of adverse childhood experiences [11]. International organisations such as the World Health Organisation, UNICEF and the World Bank, in advocating that every jurisdiction and every country in the world invest in early childhood, have developed and are supporting the widespread dissemination of the Nurturing Care Framework [12]. This provides the rationale for a focus on early childhood development, and articulates practical implementation strategies for individual countries.

It is challenging to create designated training positions in child development and community pediatrics, where management of developmental and behavioural issues is a major focus. Many will argue that with limited health budgets, the focus needs to be on staffing hospitals and looking after sick children. There is inevitably a tension between service needs and training needs [1, 13, 14]. Nevertheless in the USA [15, 16], Israel [17–19] and Australia [20, 21] there have been calls for new models of child health care delivery that better meets the contemporary needs of children and families and the evolving patterns of childhood morbidity. In Israel, Goshen—a collaborative initiated by pediatricians and now a multidisciplinary centre [22]—provides national leadership in training and professional development in the content areas that pediatricians need to practice with confidence and competence. Goshen also is working closely with a range of stakeholders on a range of initiatives designed to support the notion that child health in the twenty-first century has evolved far beyond the traditional, hospital-based model of treating illness.

Two years ago in Israel, a diverse group came together under the auspices of the Israel National Institute for Health Policy Research for an international workshop [23] to discuss the future of community child healthcare. The 62 attendees and participants included policymakers and opinion leaders from all four health funds, the Israel Pediatric Association, The Israel Ambulatory Pediatric Association, the Israel Scientific Council, the Israel National Council for the Young Child and Goshen. The topics included training in community pediatrics, the need for academic recognition, research, collaborative models of care, and so on. It was suggested that it was important to establish community pediatrics as a specialty in its own right and to provide robust academic underpinnings which would enhance training and provide role models for younger pediatricians and trainees. A detailed and comprehensive list of recommendations was developed and supported [23].

The authors of the paper (1) concluded that their findings ‘…*demonstrated a clear need to strengthen the competence of pediatricians in areas of child development*…’ and hoped that their data would raise alarms that would lead to change. Pediatricians who understand child development are in an ideal position to support children and their families, detect emerging problems at an early stage, plan appropriate interventions, reduce referrals to scarce and overburdened tertiary services, and help mobilise communities in a shared mission of optimising the health and development of all children. There have been repeated calls for changes in training and service delivery in Israel, based on good data and the expressed needs of Israeli pediatricians who lament how poorly their training has prepared them for clinical practice. A consensus set of recommendations has emerged from the recent international workshop [23] which provide a roadmap for what needs to be done. Pediatric morbidity patterns have changed dramatically in the last half century, and pediatric training and service delivery needs also to change. It is time.
